# Identification of autosomal dominant lateral temporal epilepsy caused by a novel mutation in *RELN* in China: a case report

**DOI:** 10.1186/s42494-024-00179-y

**Published:** 2024-09-10

**Authors:** Yan Chen, Yanmei Zhu, Wenqiang Zhong, Jia He, Haiyan Gou, Yulan Zhu

**Affiliations:** Department of Epilepsy Center, The Second Affiliated Hospital of Harbin Medical University, No.246 Xuefu Road, Nangang District, Harbin City, 150001 Heilongjiang Province China

**Keywords:** *RELN*, ADLTE, Familial temporal lobe epilepsy, Genetics, Glioma

## Abstract

**Background:**

Temporal lobe epilepsy is the most common type of focal epilepsy, but hereditary factors are usually overlooked. Reelin (*RELN*) is considered to be the second most common pathogenic gene implicated in autosomal dominant lateral temporal epilepsy (ADLTE). However, this mutation is not frequently discovered in the Chinese population. Additionally, there are few clinical studies regarding the connection between *RELN* and glioma.

**Case presentation:**

The healthcare records of an 8-year-old child who experienced generalized tonic-clonic seizures (GTCS) during sleep for 7 years were retrospectively analyzed. In addition to experiencing his first seizure at the age of one, his mother also suffered from GTCS during her pregnancy, and a glioma was discovered. An investigation involving gene sequencing was conducted on the proband and his parents. He was diagnosed with ADLTE once a missense mutation in *RELN* (c.1799 C > T) was identified as the causal factor. The mutation was inherited from his mother. He was taking levetiracetam (500 mg twice a day) to avoid seizures, but his mother died of status epilepticus caused by glioma recurrence two years earlier.

**Conclusions:**

Genetic issues should be given more consideration in cases of temporal lobe epilepsy. If the source of the seizures is determined to be inherited, anti-seizure medications should be used for prolonged periods. Furthermore, more research is required to determine whether mutations in *RELN* are related to the occurrence and progression of gliomas.

## Background

Temporal lobe epilepsy (TLE) is the most prevalent type of focal epilepsy [1]. Nonetheless, the genetic characteristics of TLE patients are often overlooked. A report in 1895 described clinical signs in a family across four generations, marking the first occurrence of familial temporal lobe epilepsy (FTLE) [2]. Then, in 1994, FTLE was first noted in twins [3], which launched the first hereditary TLE research. The following year, FTLE was reported in nontwin patients [4]. Autosomal dominant lateral temporal epilepsy (ADLTE) and family mesial temporal lobe epilepsy (FMTLE) are the two syndromes that comprise FTLE [5].

ADLTE was first identified in 1995, with an onset age range of 1 to 60 [4–6]. A characteristic of ADLTE is the auditory aura, which is typically linked to simple sounds like voices, but can also occasionally be linked to complex sounds like music. Another name for ADLTE is autosomal dominant partial epilepsy with auditory features (ADPEAF). Similar to mesial TLE, 10% of patients have déjà vu and dread, and 90% of patients have focal to bilateral tonic-clonic seizures, which typically happen at night. It is rare for individuals to have a history of febrile seizures, and most brain magnetic resonance imaging (MRI) scans show normal results. Electroencephalography (EEG) has consistently been used to detect abnormal discharges in the left hemisphere [7]. Leucine-rich glioma inactivating gene 1 (*LGI1*), reelin (*RELN*), and molecules interacting with casL 1 (*MICAL-1*) are the three most related genes to ADLTE studied so far. The primary pathogenic gene, *LGI1*, has a mutation in 50% of ADLTE families. These mutations were initially discovered in 2002 [8] and are situated on 10q22–24. The molecular relationship between these mutations remains unclear, as encoded protein is not an ion channel. *RELN*, situated on 7q22.1, is the second most often altered gene in ADLTE, encoding a large secretory protein. Protein misfolding brought on by mutations modifies the production of the protein and lowers its blood level, resulting in functional defects [9].* RELN* and *LGI1* have similar expression patterns in the cerebral cortex and hippocampus, suggesting that they act coordinately [9].

Here, we describe a patient with ADLTE who carries a new missense mutation in *RELN* (c.1799C > T), a mutation that is uncommon in Chinese populations. This mutation was inherited from the proband's mother. The proband suffers from ADLTE rather than a common temporal lobe seizure, and would experience seizures again if he stopped taking the anti-seizure medications. His mother experienced her first seizure at the age of one, was diagnosed with glioma during her pregnancy, and ultimately passed away from a glioma recurrence. It suggests that more research and confirmation are needed to determine whether *RELN* is also connected to the occurrence and progression of gliomas.

## Case presentation

On November 11, 2019, an 8-year-old child who had experienced paroxysmal seizures for the past seven years was hospitalized at the epilepsy center. His first convulsion happened when he was one year old, following a fever of 38.4 °C. After about a minute, his limbs became stiff and rigid, and his mouth closed involuntarily. For the next four years, he experienced similar convulsions accompanied by low-grade fever (37.2 °C–37.5 °C) without aura. When the patient was four years old, he was prescribed 250 mg of levetiracetam twice a day, and he stopped having seizures after that. Around the time the proband turned six years old, levetiracetam was gradually discontinued. After that, even without any apparent triggers, the patient frequently stumbled. In 2021, the patient had six further convulsions while sleeping; three of these happened after a low-grade fever, and other three times were afebrile. The proband's neurodevelopmental milestones were unremarkable, and he performed well in his studies. Neurological examination revealed no abnormalities. The brain MRI (3.0 T) of the proband was normal. The 24-h video-EEG data showed occasional sharp waves of moderate amplitude in the left temporal lobe (Fig. 2 a, b). The findings of other tests, such as routine blood and urine tests, biochemical and rheumatic immunity panels (to screen for autoimmune causes), and homocysteine levels (to screen for metabolic causes), were normal.

**Fig. 1:**
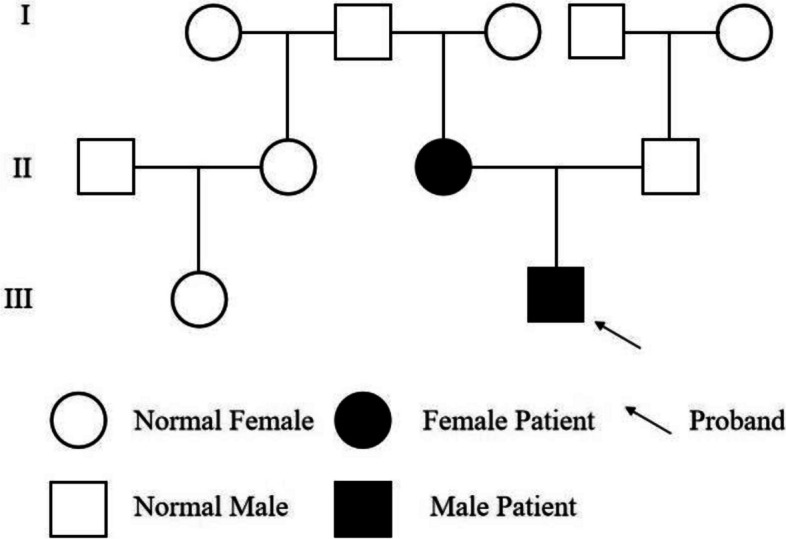
Family pedigree

According to a review of the patient's family history (Fig. 1), the patient's mother had experienced similar symptoms. She had her first seizure at the age of one, a second during her pregnancy, and a third while working at night. An MRI revealed a glioma in her left brain, which was surgically excised. His mother started having seizures again five years later. Her doctor prescribed 500 mg of sodium valproate twice a day, and she stopped having seizures afterwards.

**Fig. 2:**
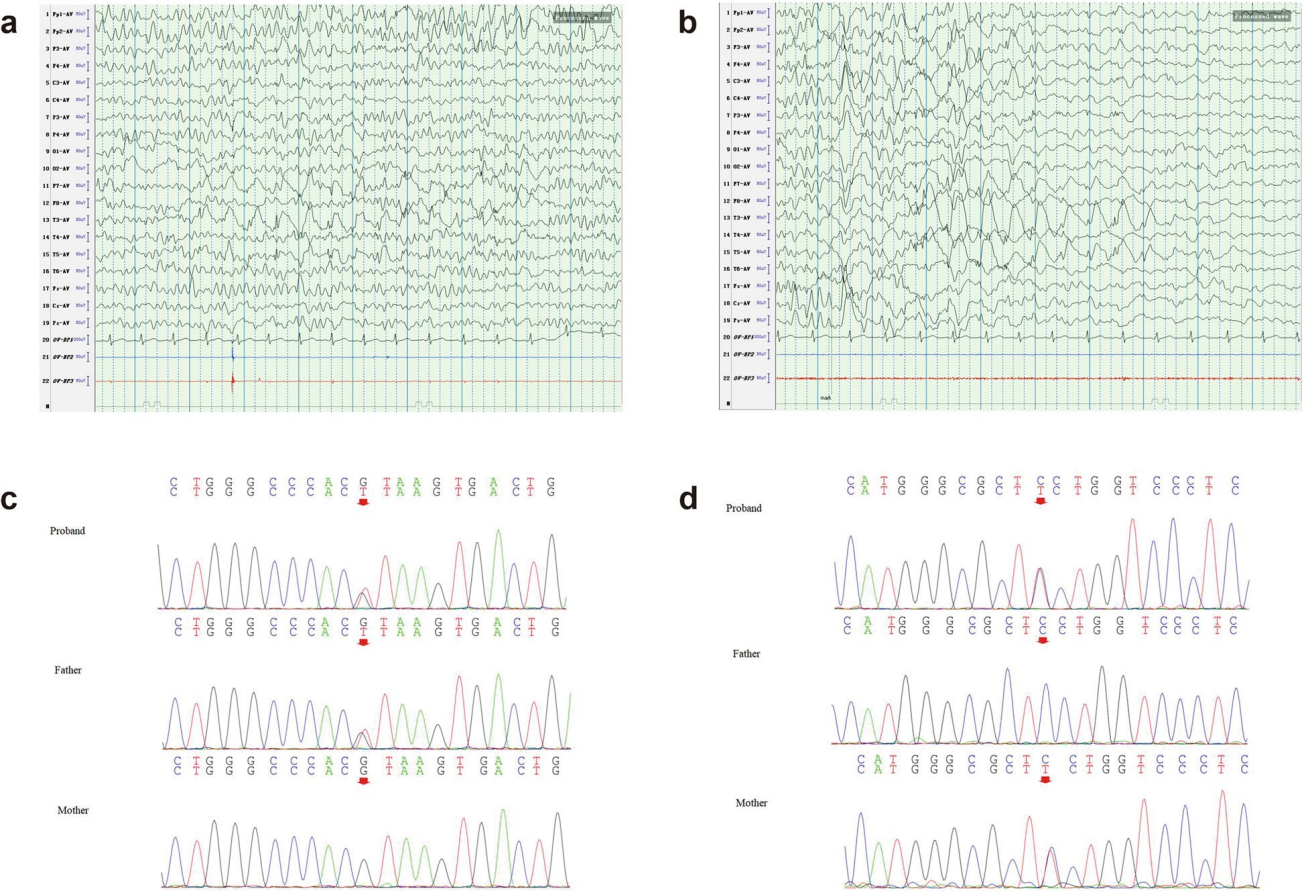
The clinical data of the proband. **a**. Sharp waves of moderate amplitude, sometimes continuously distributed, were observed in the left anterior and middle temporal lobes while awake. **b**. Sharp waves of moderate amplitude, sometimes continuously distributed, were also observed in the left anterior and middle temporal lobes while asleep. **c**. c.136+1G>T mutation in the *GAL* gene was detected in both the proband and his father. **d**.The c.1799C>T mutation in the *RELN* gene was present in both the proband and his mother

The proband underwent next-generation DNA sequencing, while his mother and father underwent validation analyses. Peripheral blood samples (5 ml from the proband and 2 ml from each parent) were collected after informed consent was obtained, and these samples were sent to Beijing Kangso Medical Inspection for genetic testing related to epilepsy (a total of 1741 genes). The findings indicated that two gene variants associated with FTLE were likely to exist (Fig. 2 c, d). The proband and his father were found to carry the 136 + 1G > T mutation in the galanin (*GAL*) gene (Table 1), while the proband and his mother were found to carry the 1799C > T mutation in *RELN* (Table 2), with the latter identified as the proband's pathogenic gene.

**Table 1:** The results of DNA sequencing, c.136 + 1G > T mutation in the *GAL* gene was detected in the proband and his father

*GAL (NM_015973)*	
Chromosomal location of the gene	chr11:68453117
Nucleotide variation	c.136 + 1G > T
Amino acid change	−
Exon/intron	Intron3
Variation type	Heterozygote
Father	Heterozygote
Mother	Not found
Frequency of variation	
ExAC	Not included
ESP6500	Not included
1000 Genomes	Not included
1000 Genomes (Han Chinese in Beijing)	Not included
1000 Genomes (Southern Han Chinese)	Not included
Kangso Health	Not included

**Tabel 2:** The results of DNA sequencing, c.1799C > T mutation in the *RELN* gene was present in the proband and his mother

*RELN (NM_005045)*	
Chromosomal location of the gene	chr7:103292201
Nucleotide variation	c.1799C > T
Amino acid change	p.Ser600Phe
Exon/intron	Exon15
Variation type	Heterozygote
Father	Not found
Mother	Heterozygote
Frequency of variation	
ExAC	0.011
ESP6500	0.011
1000 Genomes	0.006
1000 Genomes (Han Chinese in Beijing)	0.0
1000 Genomes (Southern Han Chinese)	0.0
Kangso Health	0.001

From then on, the proband, who weighed 60 kg, was treated with 500 mg of levetiracetam twice a day. After four years of follow-up, there was no recurrence of seizures, and his weight increased from 60 kg to 80 kg. He continued to take 500 mg of levetiracetam. Sadly, two years ago, the proband's mother passed away from status epilepticus caused by a glioma recurrence.

## Discussion

The proband's convulsions and the aberrant temporal lobe discharges observed on the EEG clearly supported the diagnosis of epilepsy. We ruled out the possibility that structural, metabolic, or immunological factors were the source of the seizures because there was no abnormal findings on the brain MRI, blood test markers were normal, and there were no indications of deteriorating memory or cognitive function. The later seizures could not be classified as febrile seizures since they were not associated with a rise in temperature, unlike the first five seizures, which occurred following a low-grade fever. We hypothesized that genetics might be related to the proband's seizures.

It's interesting to note that the proband carried two most likely pathogenic gene changes: a mutation in *GAL* inherited from his father and a mutation in *RELN* inherited from his mother. Both mutations could cause familial temporal lobe epilepsy, but it would be challenging to pinpoint which one is pathogenic. According to the guidelines of the American College of Medical Genetics and Genomics (ACMG), the mutation (c.136 + 1G > T) in *GAL* was likely pathogenic. Functional analysis of the mutant galanin peptide indicated that it impairs galanin function in a dominant manner, but the proband's father, grandfather, or other father relatives had no history of seizures. We hypothesized that there might be a compensatory or remedial mechanism, such as enhanced translation or protein modification. The mutation in *RELN* is classified as having unclear clinical significance based on the ACMG recommendations. However, compared to Southern Han Chinese persons in the 1000 Genomes Project and Han Chinese individuals in Beijing, the prevalence of *RELN* mutations in normal individuals ranged from 0.0 to 0.011. The findings indicate that the *RELN* missense mutation (c.1799C > T) is extremely harmful to the Chinese population. Both the proband and his mother experienced their first seizure at the age of one and responded well to anti-seizure medications. These factors lead us to believe that the pathogenic cause in the proband is the missense mutation (c.1799C > T) in *RELN*, which inherited from his mother.

Literature related to ADLTE states that a history of febrile seizures is uncommon, seizures usually evolve from focal to bilateral tonic-clonic during sleep, and they can happen between 1 and 60 years of age, with hippocampal sclerosis being rare. Patients with *RELN* mutations are more likely than those with *LGI1* mutations to experience abnormal discharges in the left temporal lobe. All of the patient’s clinical characteristics were consistent with these findings, except for the presence of the auditory aura. However, since all of the patient’s seizures occurred while he was asleep, it was challenging to identify the aura, especially in a child. Furthermore, the proband developed a recurrence following the cessation of treatment, but could be seizure-free with a low dose of anti-seizure medication. We discovered that a missense mutation in *RELN* was the cause of the seizures.

We were particularly interested in the proband's mother's history of glioma and epilepsy. His mother experienced her first seizure at the age of one, and a glioma was discovered during her pregnancy. After being treated with 500 mg of sodium valproate twice a day, she stopped having seizures. As a result, we concluded that a mutation in *RELN*, rather than only the glioma, was the cause of the proband's mother's seizures. *LGI1* is closely related to glioma. According to double immunofluorescence and confocal imaging tests, both *LGI1* and *RELN* are strongly expressed in the hippocampus and cortical neurons of adult rats, [10], indicating that these two proteins may function via the same mechanism. Additionally, studies have shown that the development and migration of glioblastomas are modulated by *RELN* signaling [11]. It is unknown whether the proband's mother's history of glioma and epilepsy was caused by a mutation in *RELN*; more research is needed to determine whether glioma and epilepsy share a common pathway. Sadly, the proband's mother passed away, we were unable to obtain an MRI or EEG of her brain.

## Conclusions

Genetic factors are often overlooked in patients with temporal epilepsy. In ADLTE, anti-seizure medications should be taken for a longer period of time than in common temporal epilepsy. This case also raised the possibility that *RELN* mutations and gliomas may be connected. It is necessary to investigate the potential mechanisms.

## Data Availability

Not applicable.

## Abbreviations

ADLTE: Autosomal dominant lateral temporal epilepsy
ADPEAF: Autosomal dominant partial epilepsy with auditory features
EEG: Electroencephalography
FMTLE: Family mesial temporal lobe epilepsy
FTLE: Familial temporal lobe epilepsy
GAL: Galanin
LGI1: Leucine-rich glioma inactivated 1
MICAL-1: Molecule interacting with casL 1
MRI: Magnetic resonance imaging
RELN: Reelin
TLE: Temporal lobe epilepsy
